# Development of an algorithm for ethnicity recording in cohorts from the UK Clinical Practice Research Datalink primary care and linked Hospital Episode Statistics databases

**DOI:** 10.1136/bmjopen-2025-100533

**Published:** 2025-07-18

**Authors:** Suhail I Shiekh, Rachael Williams, Eleanor L Axson

**Affiliations:** 1 Leicester Real World Evidence Unit, Diabetes Research Centre, University of Leicester, Leicester, England, UK; 2 Medicines and Healthcare Products Regulatory Agency, London, England, UK

**Keywords:** PUBLIC HEALTH, Electronic Health Records, EPIDEMIOLOGY, Observational Study, Primary Care

## Abstract

**Abstract:**

**Objective:**

To evaluate various prioritisation strategies within an algorithm designed to ascertain the most likely ethnicity and create a standardised methodology to benefit future research.

**Design:**

Retrospective cohort study.

**Setting:**

The Clinical Practice Research Datalink (CPRD) primary care and linked Hospital Episode Statistics (HES) data sets.

**Participants:**

The population of 54 029 174 patients included all acceptable patients registered at English practices in CPRD GOLD or CPRD Aurum from the May 2023 to May 2022 builds, respectively.

**Primary outcome measure:**

Ethnicity data within CPRD and HES data sets were identified by employing established code lists and subsequently categorised into broader ethnic groups. Changes were made to a previously used algorithm to assess their effect on ethnic categorisations. Modifications included prioritising primary over secondary care data, recent over frequent records and ‘non-other’ ethnicity categories. Different data sources were examined: CPRD with all HES data sets, CPRD with HES Admitted Patient Care (APC) only, CPRD only and HES APC only. Ethnic distributions from these variations were compared using counts and percentages, evaluating inter-rater reliability using Cohen’s kappa. Sensitivity analyses included repetition using only currently registered patients and after removing cases with unknown ethnicity. Ethnic distributions were compared with English Census 2021.

**Results:**

There was almost perfect agreement in ethnicity distributions whether prioritising primary over secondary care data (kappa=1.0000, SE=0.0001), whether prioritising most frequently or most recently recorded data (kappa=0.9824, SE=0.0001) and whether prioritising ‘non-Other’ categories (kappa=0.9705, SE=0.0001). There was moderate agreement in ethnicity distributions when sourcing data from single data sources (CPRD only (kappa=0.5554, SE=0.0001) or HES APC only (kappa=0.5526, SE=0.0001)) compared with combined data sources (CPRD and HES datasets).

**Conclusions:**

All variations of the algorithm produced similar population-level ethnicity distributions. Versions using data from multiple sources had higher inter-rater reliability than those using a subset of sources; however, there was little difference in categorisations produced by varying the hierarchical decision-making of the ethnicity algorithm. The CPRD population was representative of the English population in terms of ethnicity. While researchers should remain vigilant of the limitations of using these data, the CPRD Ethnicity Records provide a standardised and pragmatic approach to ascertaining ethnicity for future research.

**STRENGTHS AND LIMITATIONS OF THIS STUDY**

- Clinical Practice Research Datalink (CPRD) data are broadly representative of the English population in terms of age, sex, deprivation and ethnic distribution.
- CPRD and Hospital Episode Statistics (HES) data were not collected for research purposes and are based on real world data collected at primary and secondary care healthcare facilities.
- Linked ethnicity data from HES was not available for all patients; the amount of ethnicity-related codes could be affected by the amount of data available for a patient and from which data sources that data was sourced.

## Background

 The Clinical Practice Research Datalink (CPRD)^1^ collects anonymised electronic health records from primary care facilities across the UK. It produces two primary databases: CPRD GOLD, gathering data from the Vision software system, and CPRD Aurum, sourcing data from EMIS Web. Both Vision^2^ and EMIS^3^ are software systems employed by general practices to manage patient records. CPRD GOLD and CPRD Aurum can be used either independently or in conjunction. Through linking primary care data from CPRD with other patient-level data sets,^4^ such as Hospital Episode Statistics (HES),^5^ a comprehensive overview of health trends can be obtained. CPRD data are broadly representative of the English population in terms of age, sex, deprivation and ethnic distribution.^6 7^

The investigation into health disparities among different ethnicities has received extensive attention and remains a top priority for epidemiological and healthcare research.^8^,^14^ Precise and reliable data on ethnicity plays an important role in understanding these inequalities and shaping healthcare services to cater to the specific requirements of underserved ethnic groups.

Ethnicity data is recorded in the CPRD GOLD and CPRD Aurum primary care databases. Ethnicity data may also be obtained via patient-level secondary care data linked to CPRD from the HES data sets^4^—including HES Admitted Patient Care (HES APC) data, HES Outpatient (HES OP) data, HES Accident and Emergency (HES A&E) data and the HES Diagnostic Imaging Data set (HES DID)—for patients in England.

Our previous study^6^ evaluated the completeness and representativeness of ethnicity recording in CPRD and HES. This study found in CPRD-HES, 64.1% of all acceptable patients had a usable ethnicity recorded, which increased to 82.0% for currently registered patients. Many patients have multiple ethnicity records within primary and/or secondary care. Our previous study found that, for currently registered English patients, 79.3% had an ethnicity record in both CPRD and HES.^6^ Therefore, researchers must determine which ethnicity is most appropriately assigned to a patient using algorithms.

This study aimed to evaluate different prioritisations within an algorithm for ascertaining most likely ethnicity using CPRD and linked HES data to create a standardised method for obtaining and ascertaining ethnicity from these data sources. The results of this study informed the creation of new data sets, the CPRD Ethnicity Records,^15 16^ which comprise a single derived ethnicity category for each patient in CPRD GOLD and CPRD Aurum. These data sets were launched by CPRD on 24 May 2023. This paper presents the ethnic group categorisations using different prioritisations and quantifies the agreement across these algorithms to enable researchers to make more informed decisions while using ethnicity as a variable in their studies.

## Methods

### Study populations

This was a retrospective cohort study. The study population consisted of all acceptable patients registered at an English practice in CPRD GOLD from the May 2023 build^17^ and CPRD Aurum from the May 2022 build.^18^ Acceptability was defined as having research quality data as per CPRD data quality checks.^6^ There were no restrictions placed on the dates of follow-up, linkage eligibility or ethnicity recording. The populations of CPRD GOLD and CPRD Aurum were assessed together. Practices with records in CPRD GOLD and CPRD Aurum were assessed using their CPRD Aurum records only based on the deduplication information in the CPRD-provided bridging file.

### Ethnicity recording and classification

Ethnicity records in CPRD GOLD, CPRD Aurum and HES data sets were identified using previously published code lists and classified into higher ethnic categories as previously published.^6^ Briefly, ethnicity in CPRD GOLD is recorded using Read codes, ethnicity in CPRD Aurum is recorded using Systematized Nomenclature of Medicine Clinical Terms (SNOMED-CT) codes, and ethnicity in the HES data sets is recorded using bespoke codes. The HES ethnicity data was obtained from HES APC,^19 20^ HES OP,^21 22^ HES A&E^23 24^ and HES DID^25 26^ data sets. The coverage for the linked data available was up to 31 March 2021 for HES APC,^19 20^ 31 October 2020 for HES OP and HES DID^21 22 25 26^ and 31 March 2020 for HES A&E.^23 24^ Individual codes were mapped to the higher-level classification of six ethnic groups, commonly used in healthcare research: ‘Asian’, ‘Black’, ‘Mixed/Multiple’, ‘White’, ‘Other’ and ‘unknown’.

### Ethnicity algorithm variations

Our previous study^6^ used an adapted algorithm for ascertaining ethnicity developed by Public Health England.^27^ This algorithm prioritised in the following order: (1) the most frequently recorded ethnicity, (2) the most recently recorded ethnicity and then (3) records from HES over records from CPRD. Barring a definite categorisation following these steps, the algorithm prioritised ethnicity based on the frequency distribution in the UK Census by selecting the ethnicity more common in the UK Census, and finally the second most frequently recorded ethnicity if the first most recorded ethnicity was ‘Other’.

We evaluated single changes to this algorithm to assess the effect of each decision on the completeness of recording and the ethnic distribution produced. First, we changed the prioritisation of secondary care data to the prioritisation of primary care data, as previous research suggested more accurate recording in primary care.^6 28 29^ Second, we changed the prioritisation of most frequently recorded to that of most recently recorded, due to increased recording of ethnicity by general practitioners (GPs) in recent years.^6 30^ Finally, we changed the prioritisation of ‘non-Other’ values to assess whether this prioritisation unduly reduced the occurrence of ‘Other’ ethnicity. Variations of the algorithm assessed are outlined in table 1.

**Table 1:** Description of ethnicity algorithm priority variations assessed

Algorithmic step	Algorithm 1* (from our previous study)^6^	Algorithm 2 (basis of the CPRD Ethnicity Records)^15^ ^16^	Algorithm 3	Algorithm 4
1	Prioritise most frequently recorded ethnicity.		Prioritise most **recently** recorded ethnicity.	Prioritise most frequently recorded ethnicity.
2	Prioritise most recently recorded ethnicity.		Prioritise most **frequently** recorded ethnicity.	Prioritise most recently recorded ethnicity.
3	Prioritise ethnicity recorded in **secondary care.**	Prioritise ethnicity recorded in primary care.		
4	Prioritise based on UK Census 2021 ethnicity distribution.			
5	Prioritise non-Other ethnicities.			**Do not** prioritise non-Other ethnicities.

Key changes from Algorithm 2 are in bold.

* In our previous study,^6^ prioritisation in step 4 was based on the Census 2011 ethnicity distribution; this was updated for these analyses to the Census 2021 ethnicity distribution.

### Ethnicity data source variations

Additionally, the use of varying data sources for ethnicity records was assessed. Data source variations assessed were: (1) CPRD GOLD/Aurum combined with all HES data sets, (2) CPRD GOLD/Aurum combined with HES APC only, (3) CPRD GOLD/Aurum only and (4) HES APC only.

### Comparison of algorithm and data source variations

Ethnic distributions generated by each variation of the algorithm and data sources were reported as counts and percentages, rounded to the nearest hundredth, along with the proportion of patients in CPRD with a non-missing/unknown record following the application of each version of the algorithm. Comparisons of the resulting ethnic distributions were assessed for inter-rater reliability using Cohen’s kappa and prespecified inter-rater reliability categorisations.^31^

### Sensitivity analyses

First, the above analyses were repeated within the currently registered population of CPRD (online supplemental material SA-1), which is valuable for interventional studies. Second, the results of each algorithm and data source were compared using Cohen’s kappa after removing observations of unknown ethnicity (online supplemental material SA-2). This was to assess whether different versions of the algorithm and/or data sources were resulting in different ethnic categorisations for patients within the known categories (eg, white vs black) as opposed to patients having a known ethnicity in one instance versus an unknown ethnicity in another (eg, white vs unknown).

### Representativeness

Finally, we compared the ethnic distributions produced by each algorithm, excluding unknown ethnicity, to the ethnic distribution of the English population according to the 2021 Census.^32^

### Patient and public involvement

There was no patient or public involvement in this research as it is methodological and designed to assist researchers in making informed decisions about data sources for their research.

## Results

### Study population

There were 54 029 174 acceptable patients in the combined CPRD GOLD (n=12 946 589, 23.96%) and CPRD Aurum (n=41 082 585, 76.04%) data set. Of these 44 398 507 (82.18%) were eligible for linkage to HES data sets. There were 26 028 365 (48.17%) males, 27 998 819 (51.82%) females and 1990 (<0.01%) patients with indeterminate sex.

### Comparison of algorithms

There was no difference in the population-level ethnicity distribution when prioritising primary care ethnicity data (Algorithm 2) over secondary care ethnicity data (Algorithm 1) when sourcing from CPRD and all HES data sets (tables2^3^; kappa=1.0000, SE=0.0001).

**Table 2:** Comparison of proportions (%) of the population within each ethnicity category from different algorithm prioritisations

Ethnic category	Algorithm 1	Algorithm 2	Algorithm 3	Algorithm 4
Asian	7.52	7.52	7.47	7.10
Black	4.21	4.21	4.13	4.03
Mixed/multiple	1.73	1.73	1.84	1.59
White	64.61	64.61	64.62	63.77
Other	0.80	0.80	0.80	2.38
Unknown	21.13	21.13	21.13	21.13

Ethnicity data was sourced from CPRD and all HES data sets for acceptable patients.

CPRD, Clinical Practice Research Datalink; HES, Hospital Episode Statistics.

**Table 3:** Inter-rater reliability of ethnicity categorisations resulting from different algorithm prioritisations against those of Algorithm 2

Base algorithm version	Comparator algorithm version	Kappa	SE
Algorithm 2	Algorithm 1	1.0000	0.0001
Algorithm 2	Algorithm 3	0.9824	0.0001
Algorithm 2	Algorithm 4	0.9705	0.0001

Ethnicity data was sourced from CPRD and all HES data sets for acceptable patients.

CPRD, Clinical Practice Research Datalink; HES, Hospital Episode Statistics.

There was almost perfect agreement in the population-level ethnicity distribution when prioritising most recently recorded ethnicity values (Algorithm 2) over most frequently recorded ethnicity values (Algorithm 3) when sourcing from CPRD and all HES data sets (tables2^3^; kappa=0.9824, SE=0.0001).

There was almost perfect agreement in the population-level ethnicity distribution when prioritising non-Other ethnicities (Algorithm 2) compared with not prioritising non-Other ethnicities (Algorithm 4) when sourcing from CPRD and all HES data sets (tables2^3^; kappa=0.9705, SE=0.0001); however, the proportion of patients classified as ‘Other’ ethnicity increased threefold.

Additionally, there was almost perfect agreement in the population-level ethnicity distributions when comparing the various algorithm combinations (online supplemental tables S1–S4).

### Comparison of data sources

There was strong agreement in the population-level ethnicity distribution when sourcing ethnicity data from CPRD and all HES data sets compared with sourcing ethnicity data from CPRD and HES APC only when using Algorithm 2 (table 4; kappa=0.8942, SE=0.0001).

**Table 4:** Inter-rater reliability of ethnicity categorisations from different data sources using the prioritisation from Algorithm 2

Base data source	Comparator data source	Kappa	SE
CPRD and all HES data sets	CPRD and HES APC only	0.8942	0.0001
CPRD and all HES data sets	CPRD only	0.5554	0.0001
CPRD and all HES data sets	HES APC only	0.5526	0.0001

Ethnicity data was sourced from CPRD and all HES data sets for acceptable patients.

APC, Admitted Patient Care; CPRD, Clinical Practice Research Datalink; HES, Hospital Episode Statistics.

There was moderate agreement in the population-level ethnicity distribution when sourcing ethnicity data from CPRD and all HES data sets compared with sourcing ethnicity data from CPRD only when using Algorithm 2 (table 4; kappa=0.5554, SE=0.0001).

There was moderate agreement in the population-level ethnicity distribution when sourcing ethnicity data from CPRD and all HES data sets compared with sourcing ethnicity data from HES APC only when using Algorithm 2 (table 4; kappa=0.5526, SE=0.0001).

The pattern of agreement between the population-level ethnicity distributions when sourcing ethnicity data from different sources was similar within all algorithms (online supplemental tables S5–S7).

### Sensitivity analyses

Sensitivity analysis (SA)-1 showed similar inter-rater reliability when comparing algorithm types within the currently registered CPRD population (online supplemental tables S8–S12). There was reduced inter-rater reliability when comparing different data sources in the currently registered CPRD population (online supplemental tables S13–S16).

SA-2 showed similar inter-rater reliability when comparing algorithm types with and without including occurrences of ‘unknown’ ethnicity (online supplemental table S17). Inter-rater reliability was improved when excluding occurrences of ‘unknown’ ethnicity when comparing different data sources (online supplemental table S18). The pattern was the same in the currently registered population (online supplemental tables S19–S20).

### Representativeness

All the algorithms produced ethnic distributions for England that were representative of the general English population according to the Census 2021^32^ when ethnicity data was sourced from CPRD and all HES data sets for acceptable patients (table 5). When ethnicity data is sourced from CPRD and HES APC alone, the ethnicity distributions remained representative of the Census (online supplemental table S21). However, when ethnicity data was sourced from CPRD only, the proportion of patients in the white ethnic group was lower, and the proportion of Asian and black ethnic groups was higher than the Census (online supplemental table S22). Similarly, when ethnicity data was sourced from HES APC only, the proportion of patients in the white ethnic group was higher, and the proportion of Asian, black and mixed/multiple ethnic groups was lower than the Census (online supplemental table S23).

**Table 5:** Comparison of ethnicity proportions (%) from different algorithm prioritisations to the 2021 Census

Ethnic category	Algorithm 1	Algorithm 2	Algorithm 3	Algorithm 4	Census 2021
Asian	9.54	9.54	9.48	9.00	9.61
Black	5.33	5.33	5.23	5.11	4.22
Mixed/multiple	2.20	2.20	2.34	2.02	2.96
White	81.92	81.92	81.94	80.85	81.05
Other	1.01	1.01	1.01	3.02	2.18

Ethnicity data was sourced from CPRD and all HES data sets for acceptable patients. Excluding unknown ethnicity. Compared with the ethnic distribution of England from the 2021 Census.^32^

When looking at the currently registered patients in the CPRD population, the proportion of patients in the white ethnic group was marginally lower compared with the Census 2021 distribution (online supplemental table S24). All the algorithms produced similar ethnicity distributions within data sources and the same trend in more/less ethnic group proportions was seen when sourcing from single data sources (online supplemental tables S25–S27).

## Discussion

There was almost perfect agreement in ethnicity distributions using all available data sources whether prioritising primary over secondary care data, whether prioritising most frequently or most recently recorded data and whether prioritising ‘non-Other’ ethnicity categories or not prioritising ‘non-Other’ ethnicity categories; however, the proportion of patients classified as ‘Other’ ethnicity did increase threefold when not prioritising ‘non-Other’ classifications. There was almost perfect agreement in ethnicity distribution when sourcing data from CPRD and all the HES data sets compared with CPRD and HES APC only. There was moderate agreement in ethnicity distributions when sourcing data from single data sources (CPRD only or HES APC only) compared with combined data sources (CPRD and HES data sets).

The algorithm in our previous study^6^ prioritised secondary care ethnicity data over primary care ethnicity data; however, further revision of the algorithm for the CPRD Ethnicity Records^15 16^ led to the prioritisation of primary care ethnicity data over secondary care ethnicity data. This was in response to previous research suggesting that primary care records may better reflect patient self-reported ethnicity than secondary care records.^6 28 29^ These analyses, however, show that there was no difference in high-level ethnicity categorisation based on which data source was prioritised.

There was fair to almost perfect agreement in comparison of data sources overall, with Cohen’s kappa ranging from 0.5440 (SE=0.0001) to 0.8942 (SE=0.0001) using data for all acceptable patients, and from 0.3643 (SE=0.0001) to 0.9102 (SE=0.0002) using data for currently registered acceptable patients. The choice of data sources used had the greatest impact on the reliability of the results generated. For instance, for all acceptable patients, the kappa statistic for the comparison of the CPRD and all HES data sets to CPRD only or HES APC data only was around 0.55 (moderate agreement), which consistently increased to around 0.89 (strong agreement) for comparison with data from CPRD and HES APC. A similar increase was observed for currently observed patients as well. This pattern is consistent with the increase in data points from additional data sources, reducing the proportion of unknown ethnicities (online supplemental tables S18–S19).

All the algorithms produced ethnic distributions for England that demonstrated the representativeness of the CPRD population to the general English population according to the Census 2021.^32^ The CPRD population had a higher proportion of black ethnicity compared with the Census and the algorithms that prioritised non-Other ethnicity had lower proportions of ‘Other’ ethnicities compared with the Census 2021.^32^ When sourcing ethnicity data from single data sources (CPRD only or HES only), the ethnic distribution of the CPRD population changed and differed more substantially from the English Census. These differences may reflect recording biases within primary and secondary care.^33^,^35^ The effect of using different data sources for ethnicity should be considered by researchers in study designs and data source selection.

We recommend that researchers make use of all available data (CPRD and HES) when examining ethnicity, for example, by using the CPRD Ethnicity Records.^15 16^ Researchers may want to consider limiting their studies to HES linkage eligible patients only, or conducting SA using these populations, if ethnicity is a key component of their research. Furthermore, we recommend researchers to use more recent data where possible, due to significantly increased ethnicity recording following financial incentives provided by Quality and Outcomes Framework^36^ for the recording of ethnicity by GPs^6 30^ from financial years 2006/2007 to 2010/2011. When reporting and writing about ethnicity, we recommend following the UK Government style guide for writing about ethnicity,^37^ where appropriate.

### Limitations

CPRD and HES data were not collected for research purposes and are based on real world data collected at primary and secondary care healthcare facilities. We used previously published code lists.^6^ Although every effort was made to include relevant codes, there remains a possibility that there might be additional ethnicity-related codes in these databases that our search strategy did not capture. Furthermore, there exist patients, and groups of patients, where ethnicity was not recorded at the point of care, or was recorded differently, and following different procedures, at different points of care.^33^ For instance, previous studies found that in HES data, ethnicity was more completely recorded in ‘White’ ethnic groups compared with ethnic minority groups.^34^ The amount of ethnicity-related codes could also be affected by the amount of data available for a patient and from different data sources, for example, older patients have had more opportunity to contribute data points, while patients with certain medical conditions might have more interaction with healthcare services and hence more ethnicity records. These codes would potentially influence the overall ethnic distribution developed by the previous and current study and might lead to residual confounding in studies where ethnicity is a covariate of interest. If measurement error is non-differential, it could lead to the measure of effect being biased towards the null hypothesis. However, if this measurement error is differential, such as due to people of the majority ‘White’ ethnic group being less likely to have ethnicity recorded,^10^ or due to minority ethnic groups declining to provide their ethnicity to avoid discrimination,^35^ the measure of effect of a study could be biased towards or away from the null hypothesis depending on the study question. It is recommended that researchers consider these factors during study design, analysis and interpretation of results.

We only assessed variations in the hierarchy of components of a deterministic algorithm in this study using only data points available in the databases. Exploring other types of algorithms, such as probabilistic algorithms, or advanced methods to impute ethnicity was beyond the scope of this study and could be a subject of future research.

In the interpretation of these results, it is important to note that at the time of conducting this study, the coverage for linked HES data available to CPRD was limited to March 2021 or earlier.^19^,^26^ This could have led to the decreased inter-rater reliability observed in currently registered patients (∼0.37 in online supplemental tables S6–S9) between ethnicity categorisations resulting from CPRD and all HES data sets and HES APC only compared with all acceptable patients (∼0.55 in table 4, and online supplemental tables S5–S7). This limitation will be mitigated as new linked data becomes available.

These analyses were limited to the highest level of ethnicity categorisation; further study is required to assess whether algorithmic prioritisation and/or ethnicity data sources have a significant effect on ethnicity categorisation at more finite levels, such as the UK Census ethnicity categories.

These analyses were conducted by combining English populations from CPRD GOLD and CPRD Aurum. Details of the ethnic representativeness of CPRD GOLD and CPRD Aurum individually have been described previously.^6^

## Conclusion

In conclusion, the results from this study show that ethnicity algorithms including an increased number of data points available from different data sources had higher inter-rater reliability than those using a subset of sources; however, there was little difference in categorisations produced by varying the hierarchical decision-making of the ethnicity algorithm. The ethnic distributions produced by all algorithms sourcing ethnicity data from both CPRD and HES demonstrated that the CPRD population was representative of the English general population, per the 2021 Census, in terms of ethnicity. While researchers should remain vigilant of the noted limitations of using these data, the CPRD Ethnicity Records provide a standardised and pragmatic approach to ascertaining ethnicity for future research.

## Supplementary material

10.1136/bmjopen-2025-100533 online supplemental file 1

## Data Availability

Data are available upon reasonable request. Data may be obtained from a third party and are not publicly available.
