# Supplementary material for: Development of an algorithm for ethnicity recording in cohorts from the UK Clinical Practice Research Datalink primary care and linked Hospital Episode Statistics databases
Source: BMJ Open. 2025 Jul 18;15(7):e100533. doi: 10.1136/bmjopen-2025-100533 (PMC12278151; doi:10.1136/bmjopen-2025-100533)
Supplement: online supplemental file 1 [file bmjopen-15-7-s001.docx]

**Supplemental Material**

Shiekh S, Williams R, Axson EL. Methods to generate an Ethnicity Record in the UK Clinical Practice Research Datalink primary care and linked Hospital Episode Statistics databases.

**Contents**

- SA-1
- SA-2
- Tables S1-S27

**Sensitivity Analysis #1**

Methods for SA-1

Currently registered in CPRD GOLD was defined as patients who did not have a record of death or leaving their GP by 28 February 2023, and their practice having submitted data to CPRD since 1 January 2023. Currently registered in CPRD Aurum was defined as patients who did not have a record of death or leaving their GP by 30 April 2022, and their practice having submitted data to CPRD since 1 March 2022. The populations of CPRD GOLD and CPRD Aurum were assessed together. Deduplication is not required for populations restricted to the currently registered population as practices may only contribute data to one database at a time.

Results of SA-1

There were 13,106,844 currently registered patients in the combined CPRD GOLD and CPRD Aurum dataset. Of these 11,772,567 (89.82%) were eligible for linkage to HES datasets. There were 6,587,637 (50.26%) males, 6,518,648 (49.73%) females, and 559 (0.00%) patients with indeterminate sex.

There was almost perfect agreement in the population-level ethnicity distributions when comparing different algorithm prioritisations in the currently registered population (Tables S8-S12).

There was fair to moderate agreement for most population-level ethnicity distributions when comparing different sources of ethnicity data within the same algorithm prioritisation in the currently registered population (Tables S13-S16).

**Sensitivity Analysis #2**

Methods SA-2

As a sensitivity analysis, we compared the results of each algorithm and data source using Cohen’s kappa after removing observations of unknown ethnicity. This was to assess whether different versions of the algorithm and/or data sources were resulting in different ethnic categorisations for patients within the known categories (e.g., White vs Black) as opposed to patients having a known ethnicity in one instance versus an unknown ethnicity in another (e.g., White vs unknown).

Results of SA-2 in the Acceptable patient population

Agreement in the population-level ethnicity distribution was similar when excluding occurrences of unknown ethnicity as when including them when sourcing from CPRD and all HES datasets (Table S17).

Agreement in the population-level ethnicity distribution was improved when excluding occurrences of unknown ethnicity as when including them using the prioritisation from Algorithm 2 (Table S18).

Results of SA-2 in the Currently Registered patient population

Agreement in the population-level ethnicity distribution was similar when excluding occurrences of unknown ethnicity as when including them when sourcing from CPRD and all HES datasets (Table S19).

Agreement in the population-level ethnicity distribution was improved when excluding occurrences of unknown ethnicity as when including them using the prioritisation from Algorithm 2 (Table S20).

**Supplemental Tables**

| **Ethnic Category** | **Algorithm 1** | **Algorithm 2** | **Algorithm 3** | **Algorithm 4** |
| --- | --- | --- | --- | --- |
| **Asian** | 7.16% | 7.16% | 7.11% | 6.92% |
| **Black** | 3.97% | 3.97% | 3.87% | 3.88% |
| **Mixed/Multiple** | 1.60% | 1.60% | 1.71% | 1.51% |
| **White** | 60.78% | 60.78% | 60.81% | 60.31% |
| **Other** | 0.94% | 0.94% | 0.94% | 1.83% |
| **Unknown** | 25.55% | 25.55% | 25.55% | 25.55% |

**Table S1. Comparison of the proportions (%) of the English population within each ethnicity category resulting from different algorithm prioritisations.** Ethnicity data was sourced from CPRD and HES APC only for acceptable patients.

| **Ethnic Category** | **Algorithm 1** | **Algorithm 2** | **Algorithm 3** | **Algorithm 4** |
| --- | --- | --- | --- | --- |
| **Asian** | 6.08% | 6.08% | 6.06% | 6.03% |
| **Black** | 3.14% | 3.14% | 3.10% | 3.12% |
| **Mixed/Multiple** | 1.31% | 1.31% | 1.36% | 1.29% |
| **White** | 41.65% | 41.65% | 41.66% | 41.54% |
| **Other** | 0.88% | 0.88% | 0.88% | 1.10% |
| **Unknown** | 46.93% | 46.93% | 46.93% | 46.93% |

**Table S2. Comparison of the proportions (%) of the English population within each ethnicity category resulting from different algorithm prioritisations.** Ethnicity data was sourced from CPRD only for acceptable patients.

| **Ethnic Category** | **Algorithm 1** | **Algorithm 2** | **Algorithm 3** | **Algorithm 4** |
| --- | --- | --- | --- | --- |
| **Asian** | 3.59% | 3.59% | 3.58% | 3.47% |
| **Black** | 2.49% | 2.49% | 2.47% | 2.44% |
| **Mixed/Multiple** | 0.97% | 0.97% | 1.01% | 0.94% |
| **White** | 45.14% | 45.14% | 45.14% | 44.90% |
| **Other** | 1.00% | 1.00% | 1.00% | 1.45% |
| **Unknown** | 46.80% | 46.80% | 46.80% | 46.80% |

**Table S3. Comparison of the proportions (%) of the English population within each ethnicity category resulting from different algorithm prioritisations.** Ethnicity data was sourced from HES APC only for acceptable patients.

| **Base Algorithm Version** | **Comparator Algorithm Version** | **Kappa** | **Standard Error** |
| --- | --- | --- | --- |
| Algorithm 1 | Algorithm 3 | 0.9824 | 0.0001 |
| Algorithm 1 | Algorithm 4 | 0.9705 | 0.0001 |
| Algorithm 3 | Algorithm 4 | 0.9545 | 0.0001 |

**Table S4. Inter-rater reliability of ethnicity categorisations resulting from different algorithm prioritisations.** Ethnicity data was sourced from CPRD and all HES datasets for acceptable patients.

| **Base Data Source** | **Comparator Data Source** | **Kappa** | **Standard Error** |
| --- | --- | --- | --- |
| CPRD and all HES datasets | CPRD and HES APC only | 0.8942 | 0.0001 |
| CPRD and all HES datasets | CPRD only | 0.5554 | 0.0001 |
| CPRD and all HES datasets | HES APC only | 0.5527 | 0.0001 |

**Table S5. Inter-rater reliability of ethnicity categorisations resulting from different data sources using the prioritisation from Algorithm 1 for acceptable patients.**

| **Base Data Source** | **Comparator Data Source** | **Kappa** | **Standard Error** |
| --- | --- | --- | --- |
| CPRD and all HES datasets | CPRD and HES APC only | 0.8915 | 0.0001 |
| CPRD and all HES datasets | CPRD only | 0.5554 | 0.0001 |
| CPRD and all HES datasets | HES APC only | 0.5474 | 0.0001 |

**Table S6. Inter-rater reliability of ethnicity categorisations resulting from different data sources using the prioritisation from Algorithm 3 for acceptable patients.**

| **Base Data Source** | **Comparator Data Source** | **Kappa** | **Standard Error** |
| --- | --- | --- | --- |
| CPRD and all HES datasets | CPRD and HES APC only | 0.8827 | 0.0001 |
| CPRD and all HES datasets | CPRD only | 0.5440 | 0.0001 |
| CPRD and all HES datasets | HES APC only | 0.5588 | 0.0001 |

**Table S7. Inter-rater reliability of ethnicity categorisations resulting from different data sources using the prioritisation from Algorithm 4 for acceptable patients.**

| **Ethnic Category** | **Algorithm 1** | **Algorithm 2** | **Algorithm 3** | **Algorithm 4** |
| --- | --- | --- | --- | --- |
| **Asian** | 10.99% | 10.99% | 10.92% | 10.44% |
| **Black** | 5.23% | 5.23% | 5.10% | 5.02% |
| **Mixed/Multiple** | 2.40% | 2.40% | 2.55% | 2.21% |
| **White** | 75.66% | 75.66% | 75.71% | 74.63% |
| **Other** | 0.97% | 0.97% | 0.97% | 2.95% |
| **Unknown** | 4.74% | 4.74% | 4.74% | 4.74% |

**Table S8. Comparison of the proportions (%) of the English population within each ethnicity category resulting from different algorithm prioritisations.** Ethnicity data was sourced from CPRD and all HES datasets for currently registered patients.

| **Ethnic Category** | **Algorithm 1** | **Algorithm 2** | **Algorithm 3** | **Algorithm 4** |
| --- | --- | --- | --- | --- |
| **Asian** | 10.77% | 10.77% | 10.68% | 10.46% |
| **Black** | 5.08% | 5.08% | 4.92% | 4.96% |
| **Mixed/Multiple** | 2.29% | 2.29% | 2.47% | 2.16% |
| **White** | 73.84% | 73.84% | 73.90% | 73.23% |
| **Other** | 1.13% | 1.13% | 1.13% | 2.28% |
| **Unknown** | 6.90% | 6.90% | 6.90% | 6.90% |

**Table S9. Comparison of the proportions (%) of the English population within each ethnicity category resulting from different algorithm prioritisations.** Ethnicity data was sourced from CPRD and HES APC only for currently registered patients.

| **Ethnic Category** | **Algorithm 1** | **Algorithm 2** | **Algorithm 3** | **Algorithm 4** |
| --- | --- | --- | --- | --- |
| **Asian** | 10.05% | 10.05% | 10.01% | 9.95% |
| **Black** | 4.57% | 4.57% | 4.49% | 4.53% |
| **Mixed/Multiple** | 2.10% | 2.10% | 2.20% | 2.04% |
| **White** | 66.50% | 66.50% | 66.52% | 66.26% |
| **Other** | 1.33% | 1.33% | 1.33% | 1.76% |
| **Unknown** | 15.45% | 15.45% | 15.45% | 15.45% |

**Table S10. Comparison of the proportions (%) of the English population within each ethnicity category resulting from different algorithm prioritisations.** Ethnicity data was sourced from CPRD only for currently registered patients.

| **Ethnic Category** | **Algorithm 1** | **Algorithm 2** | **Algorithm 3** | **Algorithm 4** |
| --- | --- | --- | --- | --- |
| **Asian** | 4.97% | 4.97% | 4.97% | 4.85% |
| **Black** | 2.91% | 2.91% | 2.89% | 2.85% |
| **Mixed/Multiple** | 1.29% | 1.29% | 1.32% | 1.25% |
| **White** | 48.99% | 48.99% | 48.98% | 48.75% |
| **Other** | 1.20% | 1.20% | 1.20% | 1.66% |
| **Unknown** | 40.65% | 40.65% | 40.65% | 40.65% |

**Table S11. Comparison of the proportions (%) of the English population within each ethnicity category resulting from different algorithm prioritisations.** Ethnicity data was sourced from HES only for currently registered patients.

| **Base Algorithm Version** | **Comparator Algorithm Version** | **Kappa** | **Standard Error** |
| --- | --- | --- | --- |
| Algorithm 2 | Algorithm 1 | 1.0000 | 0.0002 |
| Algorithm 2 | Algorithm 3 | 0.9673 | 0.0002 |
| Algorithm 2 | Algorithm 4 | 0.9526 | 0.0002 |
| Algorithm 1 | Algorithm 3 | 0.9673 | 0.0002 |
| Algorithm 1 | Algorithm 4 | 0.9526 | 0.0002 |
| Algorithm 3 | Algorithm 4 | 0.9229 | 0.0002 |

**Table S12. Inter-rater reliability of ethnicity categorisations resulting from different algorithm prioritisations.** Ethnicity data was sourced from CPRD and all HES datasets for currently registered patients.

| **Base Data Source** | **Comparator Data Source** | **Kappa** | **Standard Error** |
| --- | --- | --- | --- |
| CPRD and all HES datasets | CPRD and HES APC only | 0.9101 | 0.0002 |
| CPRD and all HES datasets | CPRD only | 0.7140 | 0.0002 |
| CPRD and all HES datasets | HES APC only | 0.3742 | 0.0001 |

**Table S13. Inter-rater reliability of ethnicity categorisations resulting from different data sources using the prioritisation from Algorithm 1 for currently registered patients.**

| **Base Data Source** | **Comparator Data Source** | **Kappa** | **Standard Error** |
| --- | --- | --- | --- |
| CPRD and all HES datasets | CPRD and HES APC only | 0.9101 | 0.0002 |
| CPRD and all HES datasets | CPRD only | 0.7139 | 0.0002 |
| CPRD and all HES datasets | HES APC only | 0.3742 | 0.0001 |

**Table S14. Inter-rater reliability of ethnicity categorisations resulting from different data sources using the prioritisation from Algorithm 2 for currently registered patients.**

| **Base Data Source** | **Comparator Data Source** | **Kappa** | **Standard Error** |
| --- | --- | --- | --- |
| CPRD and all HES datasets | CPRD and HES APC only | 0.9102 | 0.0002 |
| CPRD and all HES datasets | CPRD only | 0.7206 | 0.0002 |
| CPRD and all HES datasets | HES APC only | 0.3643 | 0.0001 |

**Table S15. Inter-rater reliability of ethnicity categorisations resulting from different data sources using the prioritisation from Algorithm 3 for currently registered patients.**

| **Base Data Source** | **Comparator Data Source** | **Kappa** | **Standard Error** |
| --- | --- | --- | --- |
| CPRD and all HES datasets | CPRD and HES APC only | 0.8890 | 0.0002 |
| CPRD and all HES datasets | CPRD only | 0.6924 | 0.0002 |
| CPRD and all HES datasets | HES APC only | 0.3857 | 0.0001 |

**Table S16. Inter-rater reliability of ethnicity categorisations resulting from different data sources using the prioritisation from Algorithm 4 for currently registered patients.**

| **Base Algorithm Version** | **Comparator Algorithm Version** | **Including ‘unknown’ ethnicity** | | **Excluding ‘unknown’ ethnicity** | |
| --- | --- | --- | --- | --- | --- |
|  |  | **Kappa** | **Standard Error** | **Kappa** | **Standard Error** |
| Algorithm 2 | Algorithm 1 | 1.0000 | 0.0001 | 1.0000 | 0.0001 |
| Algorithm 2 | Algorithm 3 | 0.9824 | 0.0001 | 0.9627 | 0.0001 |
| Algorithm 2 | Algorithm 4 | 0.9705 | 0.0001 | 0.9385 | 0.0001 |

**Table S17. Comparison of Cohen’s kappa comparing different ethnicity algorithms including and excluding occurrences of ‘unknown’ ethnicity.** Ethnicity data was sourced from CPRD and all HES datasets for acceptable patients.

| **Base Data Source** | **Comparator Data Source** | **Including ‘unknown’ ethnicity** | | **Excluding ‘unknown’ ethnicity** | |
| --- | --- | --- | --- | --- | --- |
|  |  | **Kappa** | **Standard Error** | **Kappa** | **Standard Error** |
| CPRD and all HES datasets | CPRD and HES APC only | 0.8942 | 0.0001 | 0.9431 | 0.0001 |
| CPRD and all HES datasets | CPRD only | 0.5554 | 0.0001 | 0.8960 | 0.0001 |
| CPRD and all HES datasets | HES APC only | 0.5526 | 0.0001 | 0.8995 | 0.0001 |

**Table S18. Comparison of Cohen’s kappa comparing different data sources including and excluding occurrences of ‘unknown’ ethnicity using the prioritisation from Algorithm 2 for acceptable patients.**

| **Base Algorithm Version** | **Comparator Algorithm Version** | **Including ‘unknown’ ethnicity** | | **Excluding ‘unknown’ ethnicity** | |
| --- | --- | --- | --- | --- | --- |
|  |  | **Kappa** | **Standard Error** | **Kappa** | **Standard Error** |
| Algorithm 2 | Algorithm 1 | 1.0000 | 0.0002 | 1.0000 | 0.0002 |
| Algorithm 2 | Algorithm 3 | 0.9673 | 0.0002 | 0.9601 | 0.0002 |
| Algorithm 2 | Algorithm 4 | 0.9526 | 0.0002 | 0.9424 | 0.0002 |

**Table S19. Comparison of Cohen’s kappa comparing different ethnicity algorithms including and excluding occurrences of ‘unknown’ ethnicity.** Ethnicity data was sourced from CPRD and all HES datasets for currently registered patients.

| **Base Data Source** | **Comparator Data Source** | **Including ‘unknown’ ethnicity** | | **Excluding ‘unknown’ ethnicity** | |
| --- | --- | --- | --- | --- | --- |
|  |  | **Kappa** | **Standard Error** | **Kappa** | **Standard Error** |
| CPRD and all HES datasets | CPRD and HES APC only | 0.9101 | 0.0002 | 0.9500 | 0.0002 |
| CPRD and all HES datasets | CPRD only | 0.7140 | 0.0002 | 0.9057 | 0.0002 |
| CPRD and all HES datasets | HES APC only | 0.3742 | 0.0001 | 0.8984 | 0.0001 |

**Table S20. Comparison of Cohen’s kappa comparing different data sources including and excluding occurrences of ‘unknown’ ethnicity using the prioritisation from Algorithm 2 for currently registered patients.**

| **Ethnic Category** | **Algorithm 1** | **Algorithm 2** | **Algorithm 3** | **Algorithm 4** | **Census 2021** |
| --- | --- | --- | --- | --- | --- |
| **Asian** | 9.61% | 9.61% | 9.55% | 9.30% | 9.61% |
| **Black** | 5.33% | 5.33% | 5.20% | 5.21% | 4.22% |
| **Mixed/Multiple** | 2.15% | 2.15% | 2.30% | 2.03% | 2.96% |
| **White** | 81.64% | 81.64% | 81.68% | 81.01% | 81.05% |
| **Other** | 1.27% | 1.27% | 1.27% | 2.46% | 2.18% |

**Table S21. Comparison of the proportions (%) of the English population within each ethnicity category resulting from different algorithm prioritisations, excluding unknown ethnicity, to the ethnic distribution of England from the 2021 Census** (18)**.** Ethnicity data was sourced from CPRD and HES APC only for acceptable patients.

| **Ethnic Category** | **Algorithm 1** | **Algorithm 2** | **Algorithm 3** | **Algorithm 4** | **Census 2021** |
| --- | --- | --- | --- | --- | --- |
| **Asian** | 11.46% | 11.46% | 11.41% | 11.36% | 9.61% |
| **Black** | 5.91% | 5.91% | 5.85% | 5.88% | 4.22% |
| **Mixed/Multiple** | 2.48% | 2.48% | 2.56% | 2.42% | 2.96% |
| **White** | 78.49% | 78.49% | 78.51% | 78.28% | 81.05% |
| **Other** | 1.67% | 1.67% | 1.67% | 2.07% | 2.18% |

**Table S22. Comparison of the proportions (%) of the English population within each ethnicity category resulting from different algorithm prioritisations, excluding unknown ethnicity, to the ethnic distribution of England from the 2021 Census** (18)**.** Ethnicity data was sourced from CPRD only for acceptable patients.

| **Ethnic Category** | **Algorithm 1** | **Algorithm 2** | **Algorithm 3** | **Algorithm 4** | **Census 2021** |
| --- | --- | --- | --- | --- | --- |
| **Asian** | 6.74% | 6.74% | 6.73% | 6.53% | 9.61% |
| **Black** | 4.69% | 4.69% | 4.64% | 4.58% | 4.22% |
| **Mixed/Multiple** | 1.83% | 1.83% | 1.90% | 1.76% | 2.96% |
| **White** | 84.85% | 84.85% | 84.84% | 84.40% | 81.05% |
| **Other** | 1.89% | 1.89% | 1.89% | 2.73% | 2.18% |

**Table S23. Comparison of the proportions (%) of the English population within each ethnicity category resulting from different algorithm prioritisations, excluding unknown ethnicity, to the ethnic distribution of England from the 2021 Census** (18)**.** Ethnicity data was sourced from HES APC only for acceptable patients.

| **Ethnic Category** | **Algorithm 1** | **Algorithm 2** | **Algorithm 3** | **Algorithm 4** | **Census 2021** |
| --- | --- | --- | --- | --- | --- |
| **Asian** | 11.54% | 11.54% | 11.46% | 10.96% | 9.61% |
| **Black** | 5.49% | 5.49% | 5.36% | 5.27% | 4.22% |
| **Mixed/Multiple** | 2.52% | 2.52% | 2.67% | 2.32% | 2.96% |
| **White** | 79.43% | 79.43% | 79.48% | 78.34% | 81.05% |
| **Other** | 1.02% | 1.02% | 1.02% | 3.10% | 2.18% |

**Table S24. Comparison of the proportions (%) of the English population within each ethnicity category resulting from different algorithm prioritisations, excluding unknown ethnicity, to the ethnic distribution of England from the 2021 Census** (18)**.** Ethnicity data was sourced from CPRD and all HES datasets only for currently registered patients.

| **Ethnic Category** | **Algorithm 1** | **Algorithm 2** | **Algorithm 3** | **Algorithm 4** | **Census 2021** |
| --- | --- | --- | --- | --- | --- |
| **Asian** | 11.56% | 11.56% | 11.47% | 11.24% | 9.61% |
| **Black** | 5.45% | 5.45% | 5.28% | 5.33% | 4.22% |
| **Mixed/Multiple** | 2.46% | 2.46% | 2.66% | 2.32% | 2.96% |
| **White** | 79.31% | 79.31% | 79.38% | 78.66% | 81.05% |
| **Other** | 1.21% | 1.21% | 1.21% | 2.45% | 2.18% |

**Table S25. Comparison of the proportions (%) of the English population within each ethnicity category resulting from different algorithm prioritisations, excluding unknown ethnicity, to the ethnic distribution of England from the 2021 Census** (18)**.** Ethnicity data was sourced from CPRD and HES APC only for currently registered patients.

| **Ethnic Category** | **Algorithm 1** | **Algorithm 2** | **Algorithm 3** | **Algorithm 4** | **Census 2021** |
| --- | --- | --- | --- | --- | --- |
| **Asian** | 11.89% | 11.89% | 11.84% | 11.77% | 9.61% |
| **Black** | 5.40% | 5.40% | 5.32% | 5.36% | 4.22% |
| **Mixed/Multiple** | 2.49% | 2.49% | 2.60% | 2.42% | 2.96% |
| **White** | 78.65% | 78.65% | 78.68% | 78.38% | 81.05% |
| **Other** | 1.57% | 1.57% | 1.57% | 2.08% | 2.18% |

**Table S26. Comparison of the proportions (%) of the English population within each ethnicity category resulting from different algorithm prioritisations, excluding unknown ethnicity, to the ethnic distribution of England from the 2021 Census** (18)**.** Ethnicity data was sourced from CPRD only for currently registered patients.

| **Ethnic Category** | **Algorithm 1** | **Algorithm 2** | **Algorithm 3** | **Algorithm 4** | **Census 2021** |
| --- | --- | --- | --- | --- | --- |
| **Asian** | 8.37% | 8.37% | 8.37% | 8.17% | 9.61% |
| **Black** | 4.90% | 4.90% | 4.86% | 4.80% | 4.22% |
| **Mixed/Multiple** | 2.17% | 2.17% | 2.22% | 2.11% | 2.96% |
| **White** | 82.54% | 82.54% | 82.52% | 82.13% | 81.05% |
| **Other** | 2.02% | 2.02% | 2.22% | 2.79% | 2.18% |

**Table S27. Comparison of the proportions (%) of the English population within each ethnicity category resulting from different algorithm prioritisations, excluding unknown ethnicity, to the ethnic distribution of England from the 2021 Census** (18)**.** Ethnicity data was sourced from HES APC only for currently registered patients.
