# Supplementary material for: Development of an algorithm for ethnicity recording in cohorts from the UK Clinical Practice Research Datalink primary care and linked Hospital Episode Statistics databases
Source: BMJ Open. 2025 Jul 18;15(7):e100533. doi: 10.1136/bmjopen-2025-100533 (PMC12278151; doi:10.1136/bmjopen-2025-100533)
Supplement: Supplementary file 2 [file bmjopen-2025-100533.reviewer_comments.pdf]

PEER REVIEW HISTORY

BMJ Open publishes all reviews undertaken for accepted manuscripts. Reviewers are asked to complete a checklist review form and are provided with free text boxes to elaborate on their assessment. These free text comments are reproduced below.

ARTICLE DETAILS

Title (Provisional)

The Development of an Algorithm for Ethnicity Recording in cohorts from the UK Clinical Practice Research Datalink primary care and linked Hospital Episode Statistics databases

Authors

Shiekh, Suhail I; Williams, Rachael; Axson, Eleanor L

VERSION 1 - REVIEW

**Reviewer**	**1**
**Name**	**Elwenspoek, Martha**
**Affiliation**	**NIHR CLAHRC West, CLAHRC West**
**Date**	**21-Feb-2025**
**COI**	**None**

This is a well written paper describing the development and testing of the new CPRD Ethnicity Records. As a CPRD user, I can confirm that publications like this are very useful for the CPRD community to inform them about the limitations of the ethnicity records and it may help researchers decide how to deal with ethnicity records in their studies.

I only have very minor comments:

Line 19-20: confusing wording “at an English practice”, better to say “at English practices”

Line 119: typo “defined as defined”

Line 339 and 350: IRR not introduced anywhere

I cannot comment on the appropriate use of statistics.

**Reviewer**	**2**
**Name**	**Thayakaran, Rasiah**
**Affiliation**	**University of Birmingham, Institute of Applied Health Research**
**Date**	**12-Mar-2025**

COI **None**

General Comments:

1. Maintain consistency in terms used, such as “prioritisation” vs. “prioritizing”. Choose one consistent form throughout the manuscript (preferably British English as per journal requirements).

2. Supplementary tables should be labelled as Table S1, Table S2, and so forth, rather than Table 1, Table 2, etc.

3. Writing "Sensitivity Analysis #1" is unclear; instead, it should be labelled as "SA-1," and similarly, "Sensitivity Analysis #2" should be labelled as "SA-2."

4. Original (Line 14-16): "This study evaluated different prioritisations within an algorithm for ascertaining most likely ethnicity"

Suggested improvement:

"This study evaluated various prioritisation strategies within an algorithm designed to ascertain the most likely ethnicity"

5. Original (Line 25-26): "Alterations included prioritizing primary care over secondary care data, recent records over frequent ones, and 'non-other' values."

Suggested improvement:

"Modifications included prioritising primary over secondary care data, recent over frequent records, and 'non-other' ethnicity categories."

6. Original (Line 29-30): "Sensitivity analyses included repeating with only currently registered patients, and after removing observations with unknown ethnicity."

Suggested improvement:

"Sensitivity analyses included repetition using only currently registered patients and after removing cases with unknown ethnicity."

Specific comments:

1. In method section: Clearly described overall. However, the section detailing variations of algorithms (lines 145-148) could be clarified by briefly explaining why these specific changes were hypothesized to matter.

2. Results are presented clearly and logically: Tables and kappa values are well integrated, but ensure all percentages consistently add up to 100% (including a quick check across tables for accuracy). For example: In Table 2, the total percentage for Algorithm 3 does not sum to 100%.

3. Tables are clearly presented. However, double-check the consistency and accuracy of all figures.

4. In discussion: Clearly interprets findings, but you could expand briefly on the practical implications for future epidemiological research and how researchers might practically apply these findings.

5. In discussion: Expand slightly the limitations to explicitly mention how biases (e.g., reporting bias in ethnicity data) might practically affect research outcomes.

6. Consider adding one additional limitation about the inherent variability in ethnicity data collection practices at the clinical level, reinforcing the practical challenges encountered in such research

7. It's advising you to define the thresholds for categorizing Cohen's kappa results (such as "almost perfect," "substantial," "moderate," etc.) to help readers who might not be familiar with these metrics. This explanatory information could be placed in a footnote or within your methods section to clarify the meaning of your results.

**Reviewer**	**3**
**Name**	**Persson, Rebecca**
**Affiliation**	**Boston Collaborative Drug Surveillance Program, Epidemiology**
**Date**	**05-May-2025**
**COI**	**None**

This paper's objective was to assess the effects of various algorithm and data source choices on the assigned ethnicity categories in CPRD and linked HES data. This paper was informative and thorough and will be a useful addition to the literature for users of CPRD data. My comments are, in general, about improving the clarity of the methods and results.

Line 140-141: Can the authors further clarify what "the algorithm prioritized ethnicity based on the frequency distribution in the UK Census" means? Does it mean that if the patient has "tying" records for white and Asian, the patient will be assigned white or does it mean that if the overall CPRD population has fewer Asian patients than the census then the patient with "tying' records for white and Asian will be assigned Asian?

I found Table 1 confusing. The associated text was very understandable and I only understood the Table by comparing it to the text. I encourage the authors to develop a more stand-alone/self-explanatory table or figure design.

Lines 181 – 183: I strongly recommend that that authors provide counts/proportions of patients from Aurum vs GOLD. This will give the readers the context that a large majority of patients in this study (I assume) are from Aurum.

Lines 195 – 197: Authors should consider noting that although the kappa is very high, the effect on the proportion of patients classified as "other" (the smallest group) is ~3X.

Line 211: Is there a term instead of "nearly perfect" that can use for kappa 0.89 that is slightly less than the "nearly perfect" used for kappas of 0.98 (line 192) and 0.97 (line 197). Perhaps "very high"?

Lines 215 – 221: Can the authors provide more detail on the disagreement is between single data sources and the combined data sources? Is it primarily "unknown" ethnicity vs. a "known" ethnicity or is the disagreement between "known" categories? I understand this is related to Sensitivity Analysis #2 (lines 236-240) but it would be helpful to have this information explicitly stated somewhere in the paper. This could potentially be in the results section or as a comment for the discussion around lines 269 – 271.

Line 269: I think the authors mean "sourcing data from CPRD and *all* the HES datasets compared to CPRD and HES APC only". Is that correct? If so, this clarification would be helpful for the reader.

Lines 337 – 342: Can the authors flesh this out a bit more? Are they saying that the CPRD data capture has more recent calendar time than the HES data and thus the current patients had more time for the CPRD data to capture their ethnicity? If so, this limitation would not be mitigated with new linked data (unless the data lag for linked data is drastically improved.) However, I may be misunderstanding the argument here.

I recommend not abbreviating IRR. The authors do not use the term very often and epidemiologists associate this abbreviation with incidence rate ratio.

The table titles throughout the supplements are very hard to distinguish. Please add more detail for clarity (e.g. "Sensitivity Analysis #1", only bolding the words that distinguish each tables from those around it). Also consider moving the table titles to be above the table, rather than below.

"Black" as an ethnicity should be capitalized in all instances.

VERSION 1 - AUTHOR RESPONSE

Reviewer: 1
Dr. Martha Elwenspoek, NIHR CLAHRC West, University of Bristol Medical School
Comments to the Author:
This is a well written paper describing the development and testing of the new CPRD Ethnicity Records. As a CPRD user, I can confirm that publications like this are very useful for the CPRD community to inform them about the limitations of the ethnicity records and it may help researchers decide how to deal with ethnicity records in their studies.

Thank you for your comments, we are pleased to hear you think this work will benefit researchers.

I only have very minor comments:
Line 19-20: confusing wording "at an English practice", better to say "at English practices"

Please see suggested change in revised abstract.

Line 119: typo "defined as defined"

Thank you, please see correction in revised methods.
Line 339 and 350: IRR not introduced anywhere

Thank you, these have been replaced with "inter-rater reliability" written out in the discussion and conclusion sections.

I cannot comment on the appropriate use of statistics.

Reviewer: 2
Dr. Rasiah Thayakaran, University of Birmingham
Comments to the Author:

General Comments:
1. Maintain consistency in terms used, such as "prioritisation" vs. "prioritizing". Choose one consistent form throughout the manuscript (preferably British English as per journal requirements).

Thank you, we have standardised to British English.
2. Supplementary tables should be labelled as Table S1, Table S2, and so forth, rather than Table 1, Table 2, etc.

Thank you for your suggestion, we have made the revision.
3. Writing "Sensitivity Analysis #1" is unclear; instead, it should be labelled as "SA-1," and similarly, "Sensitivity Analysis #2" should be labelled as "SA-2."

Thank you for your suggestion, we have made the revision.
4. Original (Line 14-16): "This study evaluated different prioritisations within an algorithm for ascertaining most likely ethnicity"
Suggested improvement:
"This study evaluated various prioritisation strategies within an algorithm designed to ascertain the most likely ethnicity"

Thank you for your suggestion, we have made the revision.

5. Original (Line 25-26): "Alterations included prioritizing primary care over secondary care data, recent records over frequent ones, and 'non-other' values."
Suggested improvement:
"Modifications included prioritising primary over secondary care data, recent over frequent records, and 'non-other' ethnicity categories."

Thank you for your suggestion, we have made the revision.

6. Original (Line 29-30): "Sensitivity analyses included repeating with only currently registered patients, and after removing observations with unknown ethnicity."
Suggested improvement:
"Sensitivity analyses included repetition using only currently registered patients and after removing cases with unknown ethnicity."
Thank you for your suggestion, we have made the revision.

Specific comments:

1. In method section: Clearly described overall. However, the section detailing variations of algorithms (lines 145-148) could be clarified by briefly explaining why these specific changes were hypothesized to matter.

Thank you, we have added brief explanations.
2. Results are presented clearly and logically: Tables and kappa values are well integrated, but ensure all percentages consistently add up to 100% (including a quick check across tables for accuracy). For example: In Table 2, the total percentage for Algorithm 3 does not sum to 100%.

Thank you, we have added a comment in the methods that percentages were rounded to the nearest hundredth.
3. Tables are clearly presented. However, double-check the consistency and accuracy of all figures.

Thank you, we have doubled checked these.
4. In discussion: Clearly interprets findings, but you could expand briefly on the practical implications for future epidemiological research and how researchers might practically apply these findings.

Thank you, we have attempted to make recommendations as to what researchers should consider when using ethnicity in their research.
5. In discussion: Expand slightly the limitations to explicitly mention how biases (e.g., reporting bias in ethnicity data) might practically affect research outcomes.

Please see the limitations section, where we state: "If measurement error is non-differential it could lead to the measure of effect being biased towards the null hypothesis. However, if this measurement error is differential, such as due to people of the majority 'White' ethnic group being less likely to have ethnicity recorded [10], or due to minority ethnic groups declining to provide their ethnicity to avoid discrimination [35], the measure of effect of a study could be biased towards or away from the null hypothesis depending on the study question. It is recommended that researchers consider these factors during study design, analysis, and interpretation of results."
6. Consider adding one additional limitation about the inherent variability in ethnicity data collection practices at the clinical level, reinforcing the practical challenges encountered in such research

Please see the first paragraph of the Limitations section, where we state: "Furthermore, there exist patients, and groups of patients, where ethnicity was not recorded at the point of care, or was recorded differently, and following different procedures, at different points of care [33]."
7. It's advising you to define the thresholds for categorizing Cohen's kappa results (such as "almost perfect," "substantial," "moderate," etc.) to help readers who might not be familiar with these metrics. This explanatory information could be placed in a footnote or within your methods section to clarify the meaning of your results.

Thank you, to maintain word limits, we have already cited a paper that can be referred to where these categorisations are defined, but we have added wording to highlight that the information is available at that reference [31].

Reviewer: 3
Dr. Rebecca Persson, Boston Collaborative Drug Surveillance Program
Comments to the Author:

This paper's objective was to assess the effects of various algorithm and data source choices on the assigned ethnicity categories in CPRD and linked HES data. This paper was informative and thorough and will be a useful addition to the literature for users of CPRD data. My comments are, in general, about improving the clarity of the methods and results.

Thank you for your comments, we are glad you found the paper informative.

Line 140-141: Can the authors further clarify what "the algorithm prioritized ethnicity based on the frequency distribution in the UK Census" means? Does it mean that if the patient has "tying" records for white and Asian, the patient will be assigned white or does it mean that if the overall CPRD population has fewer Asian patients than the census then the patient with "tying' records for white and Asian will be assigned Asian?

Thank you for your comment, we have added further clarification to the methods section.

I found Table 1 confusing. The associated text was very understandable and I only understood the Table by comparing it to the text. I encourage the authors to develop a more stand-alone/self-explanatory table or figure design.

Thank you for your comments, we have revised the table.

Lines 181 – 183: I strongly recommend that that authors provide counts/proportions of patients from Aurum vs GOLD. This will give the readers the context that a large majority of patients in this study (I assume) are from Aurum.

Thank you for your comments. These analyses were exclusively conducted on the combined population. We have added a section to the limitations to highlight that and also to highlight that details of the ethnic representativeness of each database individually can be found in our previous publication [6].

Lines 195 – 197: Authors should consider noting that although the kappa is very high, the effect on the proportion of patients classified as "other" (the smallest group) is ~3X.

Thank you, we have now explicitly stated this.

Line 211: Is there a term instead of "nearly perfect" that can use for kappa 0.89 that is slightly less than the "nearly perfect" used for kappas of 0.98 (line 192) and 0.97 (line 197). Perhaps "very high"?

Thank you, we have corrected this error, the term should have been "strong". We have used pre-specified terms per the cited paper [31].

Lines 215 – 221: Can the authors provide more detail on the disagreement is between single data sources and the combined data sources? Is it primarily "unknown" ethnicity vs. a "known" ethnicity or is the disagreement between "known" categories? I understand this is related to Sensitivity Analysis #2 (lines 236-240) but it would be helpful to have this information explicitly stated somewhere in the paper. This could potentially be in the results section or as a comment for the discussion around lines 269 – 271.

Thank you, we have added a comment in the discussion section, third paragraph.

Line 269: I think the authors mean "sourcing data from CPRD and *all* the HES datasets compared to CPRD and HES APC only". Is that correct? If so, this clarification would be helpful for the reader.

Yes, thank you. We have added the word "all".

Lines 337 – 342: Can the authors flesh this out a bit more? Are they saying that the CPRD data capture has more recent calendar time than the HES data and thus the current patients had more time for the CPRD data to capture their ethnicity? If so, this limitation would not be mitigated with new linked data (unless the data lag for linked data is drastically improved.) However, I may be misunderstanding the argument here.

Thank you, the gap between linked data end (March 2021) and data analysis (May 2023) was longer than usual hence the concern that reduced data could have impacted the results.

I recommend not abbreviating IRR. The authors do not use the term very often and epidemiologists associate this abbreviation with incidence rate ratio.

Thank you, we have replaced the abbreviations with "inter-rater reliability" written out.

The table titles throughout the supplements are very hard to distinguish. Please add more detail for clarity (e.g. "Sensitivity Analysis #1", only bolding the words that distinguish each tables from those around it). Also consider moving the table titles to be above the table, rather than below.

Thank you for your suggestion, as this is supplemental material, we have made minimal revisions for clarity.

"Black" as an ethnicity should be capitalized in all instances.

Thank you, we have revised to follow the UK Government recommendations for writing about ethnicity [37].

VERSION 2 - REVIEW

**Reviewer**	**2**
**Name**	**Thayakaran, Rasiah**
**Affiliation**	**University of Birmingham, Institute of Applied Health Research**
**Date**	**09-Jun-2025**
**COI**	

Thank you for your responses to my queries. I am satisfied with your clarifications and am happy to accept them all.

Reviewer **3**
Name **Persson, Rebecca**
Affiliation **Boston Collaborative Drug Surveillance Program, Epidemiology**
Date **13-Jun-2025**
COI

The authors have addressed all of my comments. This paper will be helpful for the CPRD research community.

One note for the authors' consideration: My prior comment suggesting adding proportions/counts of Aurum vs GOLD was not intended to request stratified analyses. I apologize for this miscommunication. I intended to suggest that authors add the overall counts by database to the Study Population Results paragraph. Such as "There were 54,029,174 acceptable patients in the combined CPRD GOLD (~8 million (X%)) and CPRD Aurum (~46 million (X%)) dataset."

VERSION 2 - AUTHOR RESPONSE

Reviewer: 2
Dr. Rasiah Thayakaran, University of Birmingham
Comments to the Author:

Thank you for your responses to my queries. I am satisfied with your clarifications and am happy to accept them all.

Thank you!

Reviewer: 3
Dr. Rebecca Persson, Boston Collaborative Drug Surveillance Program
Comments to the Author:
The authors have addressed all of my comments. This paper will be helpful for the CPRD research community.

One note for the authors' consideration: My prior comment suggesting adding proportions/counts of Aurum vs GOLD was not intended to request stratified analyses. I apologize for this miscommunication. I intended to suggest that authors add the overall counts by database to the Study Population Results paragraph. Such as "There were 54,029,174 acceptable patients in the combined CPRD GOLD (~8 million (X%)) and CPRD Aurum (~46 million (X%)) dataset."

Thank you for the additional clarification, we have made the change requested.
